# Atypical clinical presentation of distal renal tubular acidosis: a case report registered in Amazonas, Brazil

**DOI:** 10.1590/2175-8239-JBN-2019-0224

**Published:** 2020-04-27

**Authors:** Daniel Monteiro Queiroz, Rolando Guillermo Vermehren Valenzuela, Ana Wanda Guerra Barreto Marinho, Samanta Samara Bicharra dos Santos, Danielle Ochoa da Silva, Maykon da Silveira Dias, Lorena de Oliveira Cruz

**Affiliations:** 1 Universidade Federal do Amazonas, Hospital Universitário Getúlio Vargas, Manaus, AM, Brasil.

**Keywords:** Nephrocalcinosis, Nephrolithiasis, Acidosis, Renal Tubular, Hypokalemia, Hypokalemic Periodic Paralysis, Nefrocalcinose, Nefrolitíase, Acidose Tubular Renal, Hipopotassemia, Paralisia Periódica Hipopotassêmica

## Abstract

We report an unusual case of a 24-year-old girl with a history of recurrent hypokalemic paralysis episodes and skin lesions on the lower limbs and buttocks, both of which had an acute evolution. In subsequent investigations, the patient also had nephrocalcinosis, nephrolithiasis, hyperchloremic metabolic acidosis and persistent alkaline urinary pH. The findings were consistent with distal renal tubular acidosis as the cause of hypokalemic paralysis. Clinical findings, immunological tests and the result of skin biopsy suggested primary Sjögren's syndrome as an underlying cause. The patient developed azotemia due to obstructive nephrolithiasis. All the features presented in this case are an unusual manifestation of distal renal tubular acidosis; so far, we are not aware of a similar report in the literature.

## RESUMO

Relatamos um caso incomum de uma jovem de 24 anos com história de episódios recorrentes de paralisia hipocalêmica e lesões cutâneas em membros inferiores e nádegas, ambas de evolução aguda. Em investigações subsequentes, verificou-se que a paciente apresentava nefrocalcinose, nefrolitíase, acidose metabólica hiperclorêmica e pH urinário persistentemente alcalino. Os achados foram consistentes com acidose tubular renal distal como causa da paralisia hipocalêmica. Achados clínicos, exames imunológicos e o resultado da biópsia de pele foram compatíveis com a síndrome de Sjögren primária como causa subjacente. A paciente evoluiu com azotemia em decorrência da nefrolitíase obstrutiva. Todas as características apresentadas nesse caso são uma manifestação incomum de acidose tubular renal distal; até o momento, não temos conhecimento de um relato semelhante na literatura.

## INTRODUCTION

Type 1 renal tubular acidosis (RTA), or distal RTA (dRTA), is a renal tubular acidification disorder characterized by hyperchloremic metabolic acidosis and persistently high urinary pH. Hypokalemia has been reported in 28-53% of patients, and can rarely present as hypokalemic paralysis. The prevalence of dRTA in primary Sjögren’s syndrome (pSS) is estimated at 5% to 25%, and approximately 5% of patients develop nephrolithiasis (mainly calcium phosphate stones); while 56% of patients have significant nephrocalcinosis.^1^^,^ 2 ^,^ 3 ^,^ 4 ^,^ 5 ^,^ 6 ^,^ 7 In this study, we describe an unusual case of dRTA, secondary to pSS, which clinically manifested with palpable purpura in the lower limbs, recurrent hypokalemic paralysis and azotemia. So far, we are not aware of a similar report in the literature.

## CASE REPORT

**Figure 1:**
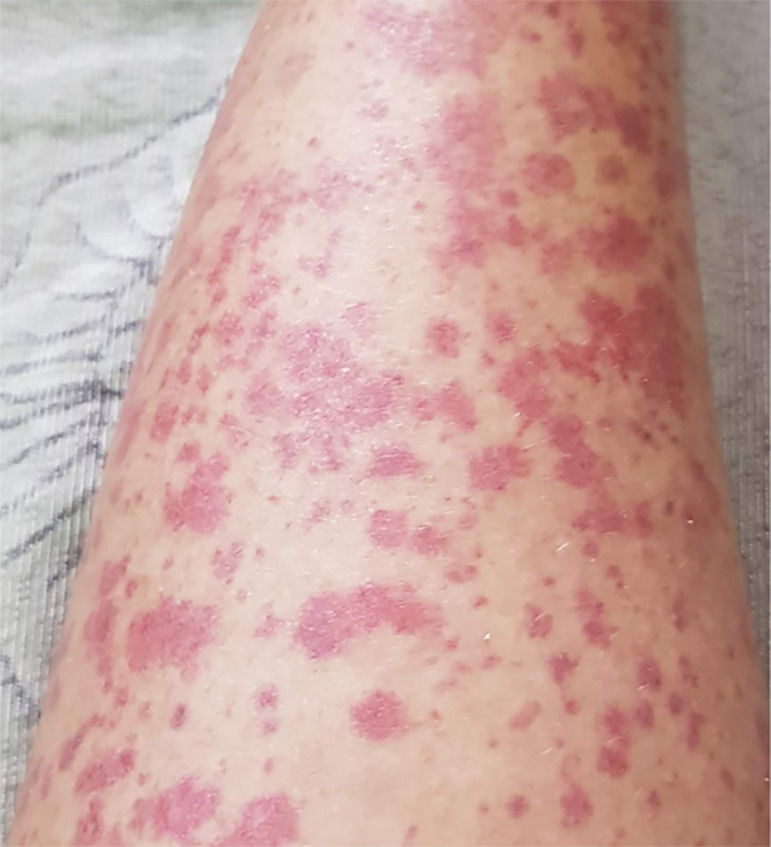
Palpable purpuric lesions of the lower limbs.

Female patient, 24 years old, healthy until August 2013, when during the night she had the first episode of weakness and difficulty in moving her limbs, also having a palpable purpura in the lower limbs and buttocks, both of acute evolution. Her condition started after an extremely exhausting and stressful day. There were no changes in the level of consciousness, sphincter release, nausea, vomiting, diarrhea, fever, arthritis, dyspnea and she did not use medications. She had no family history of a similar disease. At the time, the only documented laboratory change was hypokalemia of 1.7 mEq/L. She reported repeated hospital admissions in the past three years for the same condition mentioned above, and recurrent urinary infections. The last episode was in 2016, with a recorded potassium of 1.4 mEq/L. She received potassium replacement intravenously, followed by oral dosing for maintenance purposes, which remained asymptomatic for two years and without kidney monitoring. However, she realized that, during periods of stress, the lesions appeared on the lower limbs and buttocks, with worsening asthenia, but without impairment to daily activities. The lesions were proportional to the intensity of the stress and self-limited, with an evolution of three to four weeks. In November 2018, she was admitted to the nephrology service at the Getúlio Vargas University Hospital to clarify the clinical situation. Three weeks before this admission, she was treated for urinary infection with ciprofloxacin, when she felt severe pain in the hypogastric region, with macroscopic hematuria and elimination of a kidney stone. She denied use of non-steroidal anti-inflammatory drugs. On admission, she complained of persistent asthenia, polyuria, polydipsia, dysuria, xeroderma, xerophthalmia, xerostomia, chronic constipation, abdominal cramps and difficulty in weight gain. Recurring complaints about three years ago. In the physical exam, we noticed current weight of 45 kilos, clear-minded and oriented in time and space, without lymphadenopathy. There were no changes in her cardiovascular, respiratory and neurological exams. Her blood pressure was 120 x 80 mmHg; the heart rate was 50/min; respiratory rate 22/min; abdomen: flat, no murmur on auscultation and with mild to medium intensity pain on deep palpation on the right flank and hypogastric regions. Extremities: palpable purple lesions in the lower limbs, with greater prevalence in the malleolar region (Figure 1).

### LABORATORY FINDINGS

**Figure 2:**
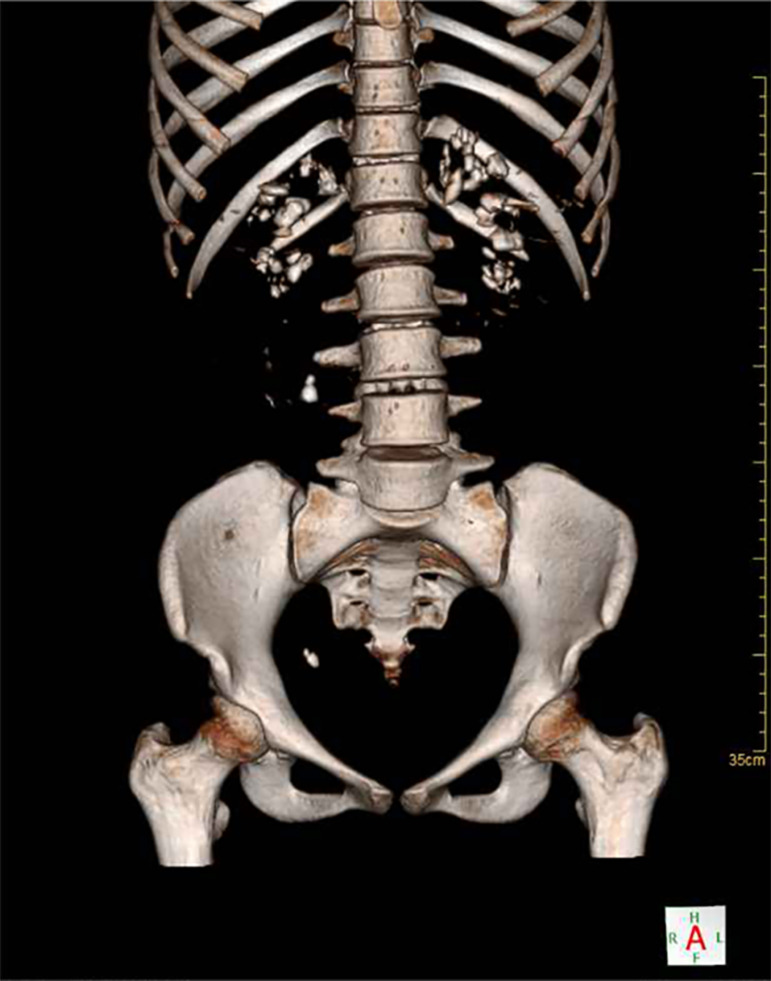
3D abdominal CT scan: bilateral nephrocalcinosis, ureteral lithiasis in the crossing of the external iliac vessels on the right, 1 cm, density 1620 uh (Hounsfield units) and ureterovesical junction.

It was evidenced through the reactive tape, persistent urinary pH 7.5, arterial blood gases with pH values = 7.164, pCO2 = 27 mmHg, HCO^3^ = 9.8 mEq / L showing hyperchloremic respiratory and metabolic acidosis. Hypokalemia (serum potassium = 3.0 mEq/L), even with oral replacement and negative urine culture. Figure 2 shows the abdominal CT scan with nephrocalcinosis and ureteral lithiasis. The diagnosis was compatible with dRTA, according to the literature.^1^^,^ 6 ^,^ 7

In the subsequent evaluation, regarding the dRTA etiology, the Schirmer test was positive in both eyes, with high titers of anti-SSA/Ro> 240.0 U/mL (normal <7.0 U/mL), anti- SSB/La > 320 U/mL (normal <7.0 U/mL) and antinuclear antibody (ANF), dotted nuclear pattern> 1: 640. On ultrasound, the glands and parotid glands were reduced and hypoechoic, with hyperechoic beams in between. The findings were conclusive for the diagnosis of pSS, according to the most recent criteria.^8^

All results of viral serology were negative, ESR and thyroid function tests were normal. The search for autoantibodies: anti-SM, anti-DNA-double helix, rheumatoid factor, anti-neutrophil cytoplasmic antibodies, complement and serum cryoglobulin tests were non-reactive. She had azotemia; urea: 61 mg/dL, creatinine: 1.34 mg/dL and normal levels of platelets, hemoglobin, blood glucose and liver function. On abdominal CT, there was bilateral hydronephrosis with an obstructive factor on the right side.

She underwent rigid ureterolithotripsy with removal of the ureteral stone and implantation of a double J catheter, confirming the cause of azotemia, and renal biopsy was not performed. Her clinical condition improved significantly after the introduction of potassium citrate 120 mEq/day, with a weight gain of 3 kg, bicarbonate of 16.7 mmol/L, and serum potassium of 4.1 mEq/L being registered on the thirtieth day of hospitalization, and normal renal function, with no evidence of proteinuria.

Two months after discharge, there were recurrences of palpable purpura in her lower limbs, with complete regression with prednisone 40 mg/day. So far, the rheumatology, urology and nephrology teams are regularly monitoring her.

## CLINICAL CASE DISCUSSION

The patient was diagnosed with dRTA secondary to PSS, clinically manifested with skin vasculitis on the lower limbs, recurrent Hypokalemic Periodic Paralysis (PPH) and obstructive azotemia due to nephrolithiasis. In dRTA, the defect is found in the interspersed alpha cells of the cortical collecting duct, whose H+ - ATPase pumps fail to secrete acid in the urine, resulting in hypokalemia, hyperchloremic metabolic acidosis and alkaline urinary pH.

Although this is a classic clinical case of dRTA with systemic metabolic acidosis (called the complete form of dRTA), it is more common in SSD to occur without systemic metabolic acidosis (called the incomplete form of dRTA). In these cases the diagnosis can only be made through urinary acidification tests.^19^

HPP is a heterogeneous disease, characterized by symmetrical muscle paralysis crises, secondary to periodic variations in potassium, which was below 2.5-3.0 mEq/L. HPP can be familial or sporadic. The clinical picture of sporadic cases is similar to that of familial cases. HPP episodes begin in the first or second decades of life, most often between 15 and 30 years of age. A diet rich in carbohydrates, salt, emotional stress, and post-exercise rest precipitate the episodes. dRTA is generally asymptomatic and can rarely present as HPP.^1^^,^ 9 ^,^ 10 ^,^ 11 ^,^ 12 Secondary causes of HPP include thyrotoxicosis (47.1%), diuretics (11.8%), dRTA, Gitelman syndrome and primary hyperaldosteronism (2.9%).^13^^,^ 14 ^,^ 15

Regarding the etiology of dRTA, the diagnosis of pSS was evident after clinical and laboratory tests, according to the most recent criteria.^8^ Sometimes systemic complications provide the first suspicion of the diagnosis, as seen in our case: muscle weakness, secondary to severe hypokalemia, palpable purpura and dRTA, leading to the suspicion of underlying autoimmune disease. Anti-SSA/Ro antibodies associated with Sjögren’s syndrome manifest in two thirds of patients, and should be evaluated in all cases. Biopsy of minor salivary glands is typically recommended to establish a diagnosis of pSS in the absence of anti-SSA/Ro antibodies. The recognition and early diagnosis of this disease are important for adequate therapeutic intervention and prevention of complications, since the treated disease has a slow course and a benign evolution. Skin biopsy was performed, which histopathological report strengthened the diagnosis of pSS, discarding other entities such as Henoch-Schönlein purple, among others. Palpable purpura is the most common dermatological manifestation associated with pSS, occurring in the lower extremities.^16^^,^ 17 Elaine L. et al.^17^ evaluated 22 patients diagnosed with pSS, with skin lesions, submitted to skin biopsies and the analysis of the main laboratory tests. The most common clinical presentation was purpura, in 10 patients (45%). The most frequent symptoms of pSS are xeroderma and skin vasculitis, and of the 30.6% who have cutaneous vasculitis, 84% manifested palpable purpura.^18^

Nephrolithiasis and nephrocalcinosis are entities frequently associated with untreated dRTA, and both are late sequelae. Acidemia promotes bone demineralization because of the mobilization of calcium phosphate from the bone. The alkaline urinary pH facilitates the precipitation of calcium and, consequently, the formation of nephrocalcinosis. In ATRd, tubular calcium reabsorption is reduced and proximal citrate reabsorption is increased, findings consistent with the patient in the case in question, according to the values found in 239mg / 24h and citraturia = 84mg / 24h, favoring the formation of kidney stones. The crystallographic evaluation of the renal calculus was performed by physical-chemical analysis (qualitative), whose presence of the composites of carbonate, oxalate and calcium phosphate.

## CONCLUSION

The diagnosis of distal renal tubular acidosis is made by clinical suspicion and laboratory findings. Early diagnosis is not easy, so subsequent investigations should be considered in any patient with hypokalemic paralysis and cutaneous manifestations of palpable purpura. As far as we know, this is the first reported case of skin vasculitis, due to primary Sjögren’s syndrome, accompanied by recurrent hypokalemic paralysis as the initial manifestation of dRTA.
